# Effects of synbiotic supplementation on gut microbiome, serum level of TNF-α, and expression of microRNA-126 and microRNA-146a in patients with type 2 diabetes mellitus: study protocol for a double-blind controlled randomized clinical trial

**DOI:** 10.1186/s13063-020-04236-y

**Published:** 2020-04-14

**Authors:** Fahime Zeinali, Seyed Mohsen Aghaei Zarch, Mohammad Yahya Vahidi Mehrjardi, Seyed Mehdi Kalantar, Alireza Jahan-mihan, Elham Karimi-Nazari, Hossein Fallahzadeh, Mahdieh Hosseinzadeh-Shamsi-Anar, Masoud Rahmanian, Mohammad Reza Fazeli, Hassan Mozaffari-Khosravi

**Affiliations:** 1 Department of Nutrition, School of Public Health, Shahid Sadoughi University of Medical Sciences, Yazd, Iran; 2 Nutrition and Food Security Research Center, Shahid Sadoughi University of Medical Sciences, Yazd, Iran; 3 Department of Genetics, Faculty of Medicine, Shahid Sadoughi University of Medical Sciences, Yazd, Iran; 4 Yazd Diabetic Research Center, Shahid Sadoughi University of Medical Sciences, Yazd, Iran; 5 Yazd Clinical and Research Center of infertility, Shahid Sadoughi University of Medical Sciences, Yazd, Iran; 6 Department of Nutrition and Dietetics, University of North Florida, Jacksonville, FL USA; 7 Department of Biostatistics and Epidemiology, Research Center of Prevention and Epidemiology of Non-Communicable Disease, School of Health, Shahid Sadoughi University of Medical Sciences, Yazd, Iran; 8 Department of Drug and Food Control, Faculty of Pharmacy, Tehran University of Medical Sciences, Tehran, Iran

**Keywords:** Type 2 diabetes mellitus, Synbiotic, Gut microbiome, Tumor necrosis factor-α, MicroRNA

## Abstract

**Background:**

The dramatic increase in the prevalence of type 2 diabetes mellitus (T2DM) is a global major challenge to health. Circulating microRNAs have been suggested as promising biomarkers for different disorders such as diabetes. Imbalances in the gut microbiome have been revealed to contribute to the progression of multiple diseases including T2DM. Recently, the consumption of probiotics and synbiotics in the treatment of various diseases has shown a substantial growth. The anti-diabetes and anti-inflammatory effects of synbiotics have been indicated, which may be due to their beneficial effects on the gut microbiome. However, further research is needed to assess the effects of synbiotics on the microbiota and their impacts on expression of microRNAs relating to T2DM. Thus, we will aim to assess the effects of synbiotics on microbiota, serum level of tumor necrosis factor-α (TNF-α), and expression of microRNA-126 and microRNA-146a in patients with T2DM.

**Methods:**

Seventy-two patients with T2DM will be recruited in this double-blind randomized parallel placebo-controlled clinical trial. After block matching based on age and sex, participants will be randomly assigned to receive 1000 mg/day synbiotic (Familact) or placebo for 12 weeks. The microRNA-126 and microRNA-146a expression levels will be measured by real-time polymerase chain reaction and serum TNF-α level will be assessed by enzyme-linked immunosorbent assay kit at the beginning and at the end of the study. Determination of the gut microbiota will be done by quantitative polymerase chain reaction methods at baseline and at the end of the trial. Biochemical assessments (glycemic and lipid profiles) will also be conducted at onset and end of the study.

**Discussion:**

This is the first randomized controlled trial that will determine the effect of synbiotic supplementation on the gut microbiota and its probable impacts on serum levels of TNF-α and expression of related microRNAs in patients with T2DM.

**Trial registration:**

Iranian Registry of Clinical Trials: IRCT20180624040228N2. Registered on 27 March 2019. http://www.irct.ir/trial/38371.

## Background

Type 2 diabetes mellitus (T2DM) is one of the most common chronic diseases and is one of the most important causes of morbidity and mortality in the world [1]. According to the International Diabetes Federation (IDF), 451 million people had T2DM worldwide in 2017, and the number is expected to reach 693 million adults by 2045 [2]. T2DM is characterized by insulin resistance [3] and chronic low-grade inflammation with abnormal production of inflammatory mediators such as tumor necrosis factor-α (TNF-α) and interleukins [4]; this is often unknown until high blood glucose levels are observed [5]. In patients with T2DM, there is an association between hyperglycemia and increased risk of microvascular complications including nephropathy, retinopathy, and neuropathy, which might damage multiple organs such as the kidneys, eyes, and nerves [6, 7]. Thus, identifying new early biomarkers for diabetes is critical to prevent further serious health problems related to T2DM.

Recent studies have shown that a group of small non-encoding RNAs, namely, microRNAs (miRNAs) can be used as novel biomarkers to detect the progression and predict the complications of diabetes in the early stages of the disease [8, 9]. The miRNAs are relatively small (about 20–24 nucleotides) endogenous RNAs that regulate gene expression by suppressing the post-transcriptional stage [10]. They play important roles in various biological processes ranging from cellular differentiation and metabolism to cancer development [11–13]; consequently, dysregulation of miRNAs is linked to many diseases [9], including T2DM [14–17]. Recent studies showed that the level of circulating microRNA-126 (miR-126) is significantly increased in patients with T2DM compared to non-diabetic people [18, 19]. Moreover, reduced concentrations of microRNA-146a (miR-146a) have been linked to a proinflammatory state related to T2DM [20, 21].

T2DM is a multifactorial disease caused by a combination of genetic and environmental factors [22, 23]. Recent studies have suggested altered composition of the intestinal microbial community as a new candidate that may cause T2DM [24, 25]. The gut microbiome refers to the more than 10^14^ bacteria living in the human gastrointestinal tract [26]. An imbalance in the microbiome is called dysbiosis, which is characterized by decreased diversity or alteration of the gut microbiota pattern, such as reduced numbers of butyrate-producing bacteria, increased abundance of opportunistic pathogens, and decreased short-chain fatty acid-producing bacteria. There is evidence showing that dysbiosis in the gut can cause numerous disease conditions including obesity, T2DM, and neurological disorders, as well as cardiovascular, fatty liver, and inflammatory bowel disease [27, 28]. The role of the microbiome in metabolic disorders may be via affecting the host’s energy balance and metabolism, immune system, inflammatory signals, and the maintenance of the normal function of the intestinal epithelial cells [29, 30]. The gut microbiota can also modulate host miRNA expression [31]. Current findings suggest that patients with T2DM have an altered gut microbiome compared with healthy subjects [32, 33]. Karlsson et al. found a reduction in butyrate-producing bacteria, including *Roseburia* and *Faecalibacterium prausnitzii*, in the gut microbiome of people with T2DM compared with healthy individuals [34]. The composition of the gut microbiome is affected by various environmental factors such as diet and drugs [35]. Due to the enormous impacts of the gut microbiota on T2DM and other chronic diseases, a range of microbiome-targeted therapeutic approaches are recently being explored. These include probiotic and prebiotic interventions, which are relatively safe, non-invasive measures in gut microbiome modulation [36–38].

Probiotics are live microorganisms that can confer beneficial effects on the host regarding the sufficiency of consumption via modulating the gut microbiota [39]. Prebiotics are non-digestible but fermentable fibers which can alter the gut microbiota composition by promoting the growth or activity of beneficial bacteria [36]. Short-chain fatty acids (SCFAs), including acetate, propionate, and butyrate, which are fermented from dietary fiber by the gut bacteria, play a critical role in energy metabolism [40]. Butyrate also has a beneficial role in maintaining the host’s intestinal integrity, leading to the prevention of endotoxemia [41]. Synbiotics are combinations of probiotics and prebiotics such that their health benefits are synergistic [42]. Therefore, it is hypothesized that a change in the intestinal microbiota induced by synbiotic supplementation will modulate expression of related miRNAs and result in improved metabolic health. A recent systematic review and meta-analysis of randomized controlled trials reported a significant change in glucose metabolism and improvement in lipid profiles from synbiotic interventions [43]. Yoo and Kim have shown that probiotics and prebiotics can affect metabolic disorders, including T2DM and cardiovascular disease, by improvement of the gut microbiome, leading to regulation of insulin-signaling and a reduction in cholesterol [44]. Nevertheless, the effects of synbiotics on the gut microbiota composition and their impacts on expression of miRNAs related to T2DM have not yet been investigated in human studies. Few clinical trials have assessed the effects of probiotics on the microbiome and miRNA expression in other chronic diseases; the majority of studies were established in animal models. Recently, one study reported that probiotic treatment in colitic mice exerted positive effects on their immune response by modulating the expression of some miRNAs, perhaps through their ability to modify the intestinal microbiota composition [45].

To the best of our knowledge, there are no clinical trials that assessed the effects of synbiotic treatment on changes of gut microflora composition and expression of miRNAs in patients with T2DM.

### Objectives

The present randomized clinical trial (RCT) aims to investigate the effects of synbiotic supplementation on microbiome composition as well as serum levels of TNF-α and expression of miR-126 and miR-146a in patients with T2DM. Secondary objectives of this trial are to compare the changes in glycemic control indices, including fasting blood sugar, glycosylated hemoglobin (HbA1c), fasting insulin, pancreatic beta cell function, insulin resistance and insulin sensitivity, as well as serum levels of lipids, namely triglycerides (TG), total cholesterol (TC), low-density lipoprotein cholesterol (LDL-C), and high-density lipoprotein cholesterol (HDL-C), between the control and intervention groups.

## Materials and methods

### Study design

**Fig. 1:**
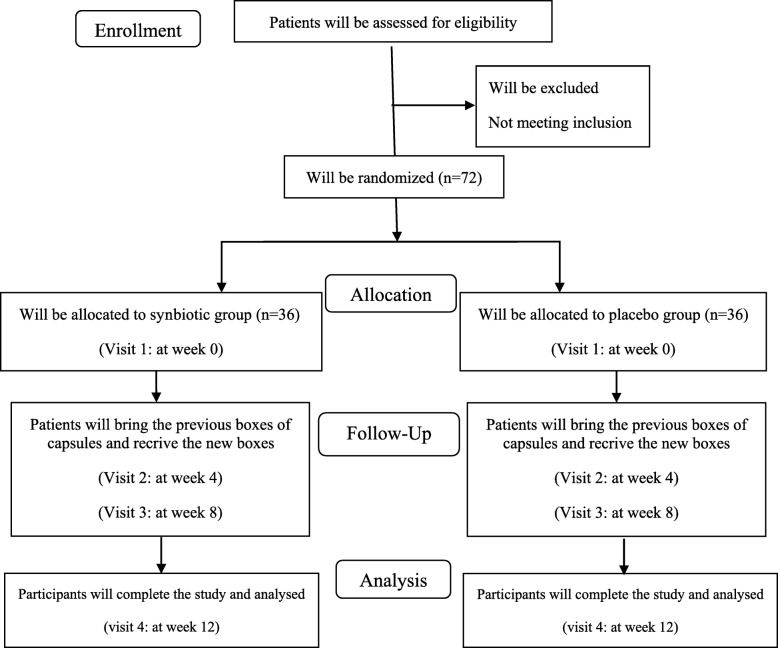
Overview of the study

**Fig. 2:**
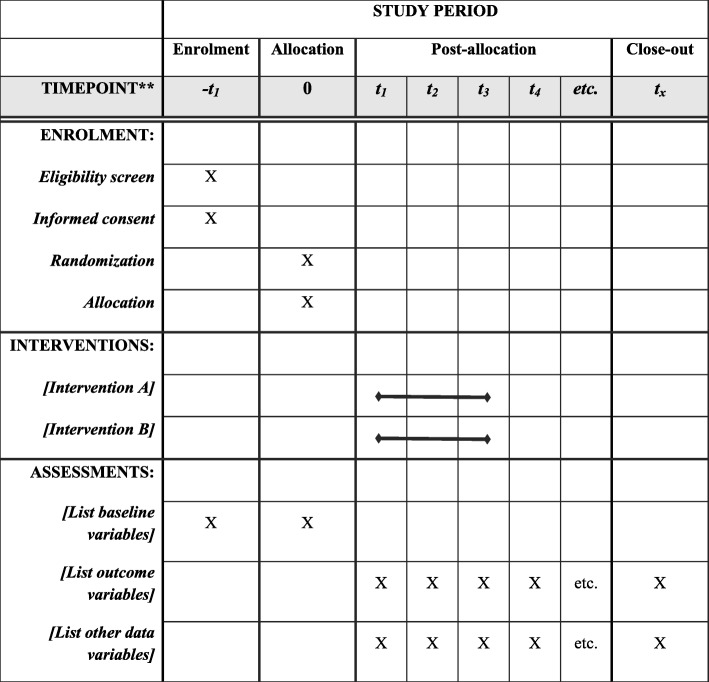
SPIRIT figure: template of the content for the schedule of enrollment, interventions, and assessments

This study is a randomized, double-blind, placebo-controlled, parallel clinical trial conducted at the Diabetes Clinic Center in Yazd, Iran. Patients with T2DM will be randomly assigned to a treatment or control group (*n* = 36/group) and will receive either a daily synbiotic (1000 mg) or placebo, respectively, for 12 weeks. An overview of the study is presented in Fig. 1. The Standard Protocol Items: Recommendations for Interventional Trials (SPIRIT) checklist was used to detail the study protocol [46] (see Additional file 1). A SPIRIT diagram including the timepoints of the study is shown in Fig. 2. Any methodological changes in the study design which could affect study procedures or participants’ safety will be discussed by the ethics committee before the study is conducted.

### Inclusion criteria

Participants will be eligible for this study if they meet the following criteria:

- Men and women who have been diagnosed with T2DM within the previous 6 months and are treated by medication for diabetes
- Age 25–65 years
- Body mass index 25–35 kg/m^2^
- 6.5% ≤ HbA1c ≤ 8.5%.

### Exclusion criteria

The following people will be excluded from the study: those using alternative therapies with hormones (insulin, corticosteroids) or vitamin supplements; those with chronic kidney, liver, or pulmonary diseases, chronic or acute inflammatory diseases (especially acute pancreatic inflammation and endocarditis), valvular heart disease, or irritable bowel syndrome; those having diabetes complications (nephropathy, cardiomyopathy, retinopathy, diabetic foot ulcers); those with low immune systems (or autoimmune disorders); pregnant or lactating females; those using tobacco or alcohol; those who have consumed probiotics or been treated with antibiotics within the past month; and those following an unusual diet up to 1 month before the study.

### Setting

The subjects’ recruitment process will be advertised through flyers distributed at Diabetes Clinic Centers in Yazd, Iran, affiliated with Shahid Sadoughi University of Medical Sciences, which is the main center in the province in the field of treatment, prevention, research, and services for patients with diabetes. Interested candidates will be invited for the screening process. Blood samples will be obtained from the volunteers, and HbA1c will be measured. Then eligible participants will be invited for their first visit, at which two trained study staff will explain to them the project protocol and procedures in detail and also provide them with an information sheet for their record. Informed consent will be obtained from those who agreed to participate in the study. Demographic information questionnaires including general data such as diseases, medications, and supplements will be administered via interviews. All questionnaires will be approved by the ethics committee of Shahid Sadoughi University of Medical Sciences. Thereafter, participants will be randomly assigned to the intervention or placebo group (*n* = 36/group). Verbal and written instructions on how to take the synbiotic or placebo capsules will be provided at the first visit and at each following visit. All participants, researchers, statistical analysts, and laboratory staff will be blind to the intervention.

### Randomization and blinding

The present study is a two-arm, double-blind parallel RCT in which participants will be randomized 1:1 using the method of stratified block randomization based on age (25–45 and 45–65 years) and sex (male and female), so that the number of samples assigned to each of the groups will be equal [47, 48]. To randomly allocate eligible individuals to the intervention and control groups, computer-generated random numbers will be used, enclosed in sealed envelopes by a third person who will not be involved in the study. Capsules containing the synbiotic and placebo are similar in shape and appearance and will be packed in the same boxes (in terms of color and shape). The capsule boxes will be labeled with codes “A” and “B” by a third party who has no direct involvement in the study. Both participants and investigators will be blinded to the content of the boxes, allocation, and study treatment until data analysis.

### Sample size

The required sample size is calculated based on data from a previous human study, which assessed the effects of probiotic yogurt consumption on inflammatory biomarkers [49] by considering the serum level of TNF-α as the primary variable. A mean difference in serum TNF-α levels of 0.84 pg/mL between the two groups is aimed to be detected for a specified α of 0.05 and a study power of 80%. Based on the proposed formula for parallel clinical trials [50], we reached a sample size of 33 participants in each group. Assuming a possible drop-out rate of 10%, 36 patients will be enrolled in each group. G*Power 3.1 software was used for the sample size calculation.

### Intervention

The intervention group will take two capsules of synbiotic per day (one capsule after lunch and one after dinner), and the control group will take two capsules of placebo (containing 500 mg lactose, magnesium stearate, and talc) per day at the same times for 12 weeks. The synbiotic and placebo capsules look and smell identical and will only be differentiated by a label “A” or “B” on the box. The boxes will be labeled by a third party who is not involved directly in this study. Each synbiotic capsule (Familact, produced by Zisttakhmir Company, Tehran, Iran) contains 500-mg levels (109 colony-forming units [CFUs]) of seven beneficial bacteria (*Lactobacillus casei* 3 × 10^8^ CFU/g, *Lactobacillus acidophilus* 2 × 10^8^ CFU/g, *Lactobacillus bulgaricus* 2 × 10^9^ CFU/g, *Lactobacillus rhamnosus* 3 × 10^8^ CFU/g, *Bifidobacterium breve* 2 × 10^8^ CFU/g, *Bifidobacterium longum* 1 × 10^9^ CFU/g, and *Streptococcus thermophilus* 3 × 10^8^ CFU/g), along with a prebiotic fructooligosaccharide (contributing to the growth and activity of probiotics) and other components (lactose, magnesium acetate, talc). Each box contains 30 capsules. Participants will be given six boxes for the entire 3-month intervention (two boxes at the beginning of each month). Moreover, patients will continue to take diabetic medication during the study. The Diabetes Clinic Center in Yazd and Shahid Sadoughi University of Medical Sciences will be responsible to follow up any reports from participants for any potential relevant issues and adverse events. Any possible adverse events will be reported to the medical ethics committee throughout the study. Participants will be informed at the beginning of the study via the consent form that they have the right to withdraw from the study at any time for any reason or even for no reason.

### Adherence

Adherence to the intervention will be monitored every day through phone interviews. Written and verbal instructions on how to take the capsules will be provided at the first visit and at each following visit. Individuals will be asked to take one capsule after lunch and one after dinner, which also will improve their adherence to the intervention. Moreover, participants will be asked to record their daily consumption of supplements or placebos in the study diary. All participants will be asked to return any remaining capsules in the boxes to the next visit. At the end of the 12-week intervention, if the number of remaining capsules for each participant is more than 10% of the total administered capsules (*n* = 18), that participant will be classified as non-adherent. All participants, both adherent and non-adherent, will follow the same schedule.

### Outcomes

The primary outcomes consist of expression of miR-126 and miR-146a, the gut microbiome status, and serum level of TNF-α. Secondary outcomes will be serum levels of lipid profiles (TG, total cholesterol, HDL-C, and LDL-C), anthropometric data, fasting plasma glucose level, HbA1c, fasting plasma insulin level, insulin sensitivity (quantitative insulin sensitivity check index [QUICKI]), homeostasis model assessment of insulin resistance (HOMA-IR), and homeostasis model assessment of beta cell function (HOMA-B). All these factors will be measured at onset and end of the study.

### Dietary intake and physical activity assessment

Participants will be requested to complete 3-day food record and physical activity record forms, including one weekend day and two weekdays at the beginning and end of the study (one in the first week and the other one in the last week of the intervention), to ensure lack of change in dietary intake and participants’ physical activity during the survey. Daily nutrient intakes will be analyzed using Nutritionist IV software (First Databank, San Bruno, CA, USA), in which the database was modified for Iranian foods. Physical activity records will be analyzed using metabolic equivalent task-hours (MET-h)/day values for every physical activity [51], regarding the time spent by each individual.

### Anthropometric assessment

Anthropometric indices will be quantified at the onset and end of the intervention. Height will be measured, with the participant at a standing position next to the wall, without shoes, to the nearest 0.1 cm with a stadiometer (Seca, Hamburg, Germany); waist and hip circumferences will be taken with minimal inspiration to the nearest 0.1 cm at the smallest waist circumference area with non-stretching tape. Waist-to-height and waist-to-hip ratios will be calculated via standard equations. Body weight will be measured to the nearest 0.1 kg, with the participant in a fasting state, without shoes, and wearing only light clothing, using a weighing calibrated scale (Seca, Hamburg, Germany). Body mass index will be calculated by dividing the body weight in kilograms by the height in meters squared.

### Blood sampling, biochemical assessment

After 12 h of fasting, a 10-mL venous blood sample will be collected from each participant at baseline and end of trial. A 5-mL blood sample will be collected in test tubes containing clot activator and centrifuged at room temperature, at 3000 rpm for 10 min (Eppendorf AG, Hamburg, Germany) for serum isolation. Serum samples will be stored at − 70 °C. The remainder of the blood sample will be collected in two tubes containing ethylenediaminetetraacetic acid (EDTA) for separate RNA extraction and HbA1c measurement. Biochemical analyses will include serum lipid profiles (TC, HDL-C, LDL-C, TG) and fasting blood glucose using the enzymatic colorimetric method by commercial kits (Pars Azmoon, Tehran, Iran). HbA1c will be measured by high-pressure liquid chromatography (Pars Azmoon, Tehran, Iran). Serum insulin and TNF-α will be assessed using an enzyme-linked immunosorbent assay (ELISA) kit. HOMA-B, QUICKI, and HOMA-IR will be calculated [52]. To keep patient confidentiality, all laboratory information will be saved using an ID number.

### Molecular measurements

MicroRNAs (miR-126 and miR-146a) will be extracted directly from whole blood using Bio Basic purification kit protocol (Bio Basic, Markham, ON, Canada). After normalization, miRNAs will be reverse transcribed to complementary DNA (cDNA) with a Bon-Mir First Strand cDNA Synthesis Kit (Bonyakhteh, Tehran, Iran) according to the manufacturer’s instructions. MiRNA gene expression will be investigated with the real-time polymerase chain reaction (PCR) method, using a Rotor Gene 6000 machine (Corbett, Concorde, NSW, Australia) with a BONmiR quantitative PCR (QPCR) Kit (Bonyakhteh, Tehran, Iran). The relative amount of miRNAs (fold change) will be calculated according to the method 2^–ΔΔCt^ (Livak) [53].

### Stool samples and microbiota assessment

Stool samples will be collected in sterile containers and will be stored at 4 °C until transport to the laboratory at onset and end of trial. DNA extraction from stool samples will be performed with the QIAamp DNA stool Mini kit (Qiagen, Hilden, Germany) following the manufacturer’s instructions. Microbial composition will be identified by QPCR and 16s ribosomal RNA (rRNA) sequencing using primers of six groups of bacteria including *Akkermansia*, *Faecalibacterium*, *Prevotella*, *Bifidobacterium*, *Lactobacillus*, and Bacteroidetes-Firmicutes.

### Statistical analysis

Statistical analyses will be performed with SPSS software V.23.0 (SPSS Inc., Chicago, IL, USA). Normality of distribution of data will be assessed by the one-sample Kolmogorov-Smirnov test. At first, the primary information of the intervention and control groups will be compared. Continuous data will be presented as means ± standard deviation (SD), and categorical data will be expressed as numbers and percentages. The independent samples *t* test and the Mann-Whitney *U* test will be used for analyzing the differences in parametric continuous and asymmetric variables between the two groups, respectively. The paired *t* test will be used to identify the effect of the intervention in each group. General linear models will be applied to analyze the effects of the synbiotic relative to placebo after adjusting the baseline factors and individuals’ characteristics. The intention-to-treat (ITT) method and the per protocol analysis will be applied for data analysis. The former considers all participants in the trial and ignores anything that happens after randomization such as misallocation and non-compliance. The latter is adjusted for actual treatment. The results of the aforementioned analyses will be compared with each other.

*P* values < 0.05 will be considered as statistically significant.

## Discussion

Type 2 diabetes mellitus (T2DM) is one of the most prevalent endocrine diseases worldwide. Serum microRNA expression is a novel biomarker to diagnose and prognose T2DM. Recent studies have demonstrated that altered composition of the gut microbiome may well be a triggering point for T2DM onset. Synbiotics are a combination of probiotics and prebiotics which could have a profound impact on the gut microbiome. Synbiotics have recently been found to have anti-diabetes effects, probably due to their beneficial effects on the gut microbiota composition. However, the effects of synbiotics on the gut microbiota and their impacts on expression of microRNAs relating to T2DM have not yet been assessed in human studies. Therefore, the present study will investigate the effects of synbiotics on the gut microbiota and expression of miR-126 and miR-146a in patients with T2DM. The results of this study will provide clinical evidence on the effectiveness of synbiotic supplementation in improving gut bacteria composition, mitigating inflammation, and improving glycemic and lipid profiles in patients with T2DM. One of the strengths of this study is that it is a double-blind randomized controlled clinical trial. If the results of this trial are valid, they will contribute to new methods for the upgraded treatment of diabetes.

## Trial status

Participant recruitment started in September 2019 and is ongoing.

## Supplementary information

**Additional file 1.** SPIRIT 2013 Checklist.

## Data Availability

The datasets generated during the current study will be available via the corresponding author on reasonable request.

## Abbreviations

BMI: Body mass index
CFU/g: Colony-forming units per gram
EDTA: Ethylenediaminetetraacetic acid
ELISA: Enzyme-linked immunosorbent assay
HbA1c: Glycosylated hemoglobin type A1c
HDL-C: High-density lipoprotein cholesterol
HOMA-B: Homeostasis model assessment of beta cell function
HOMA-IR: Homeostasis model assessment of insulin resistanceIDF International Diabetes Federation
ITT: Intention to treat
LDL-C: Low-density lipoprotein cholesterol
MET: Metabolic equivalent task
miR: MicroRNA
miRNA: MicroRNA
PCR: Polymerase chain reaction
QPCR: Quantitative polymerase chain reaction
QUICKI: Quantitative insulin sensitivity check index
RCT: Randomized clinical trial
SCFA: Short-chain fatty acid
SD: Standard deviation
T2DM: Type 2 diabetes mellitus
TC: Total cholesterol
TG: Triglycerides
TNF-α: Tumor necrosis factor-α
WC: Waist circumference
WHR: Waist-to-hip ratio
